# Catalysts of plant cell wall loosening

**DOI:** 10.12688/f1000research.7180.1

**Published:** 2016-01-29

**Authors:** Daniel J. Cosgrove

**Affiliations:** 1 Department of Biology, 208 Mueller Lab, Pennsylvania State University, University Park, PA, USA

**Keywords:** plant cell wall, cell wall expansion, wall loosening

## Abstract

The growing cell wall in plants has conflicting requirements to be strong enough to withstand the high tensile forces generated by cell turgor pressure while selectively yielding to those forces to induce wall stress relaxation, leading to water uptake and polymer movements underlying cell wall expansion. In this article, I review emerging concepts of plant primary cell wall structure, the nature of wall extensibility and the action of expansins, family-9 and -12 endoglucanases, family-16 xyloglucan endotransglycosylase/hydrolase (XTH), and pectin methylesterases, and offer a critical assessment of their wall-loosening activity

## Introduction

The growing cell wall of plants is both strong and extensible. Its mechanical strength lets it resist the tensile stresses in the plane of the wall (~10 MPa or more) generated by the internal hydrostatic pressure (turgor) typical of plant cells (~0.5–1 MPa). Its extensibility lets it expand irreversibly in surface area by 10- to more than 1,000-fold between its initial formation at cell division and the subsequent cessation of growth at developmental maturity. Such expansion involves selective wall loosening to enable irreversible extension, or “creep” (see Table 1 for explanations of biomechanical terms; see 1 for additional details of the biomechanical aspects of plant cell growth). This process enables plant cells to grow to more than 100 times the size of their meristem initials. Lacking such a process, the tallest trees on Earth would be shorter than the average reader of this article. Synthesis and incorporation of new structural components into the growing walls are also required in the long term to prevent loss of mechanical integrity, but wall synthesis in most plant cells is not linked mechanistically to expansion, as it is in bacteria. In this article, I briefly summarize current concepts of plant cell wall loosening and the proteins that catalyze it.

**Table 1.** Brief explanations of biomechanical terms often used in cell wall mechanics in the context of plant growth.

Viscoelasticity	The mechanical property of materials with both elastic and viscous characteristics. Plant cell walls are viscoelastic as a result of their polymeric structure, but they have additional, time-dependent biomechanical responses that depend on wall loosening.
Wall loosening versus remodeling	Loosening refers to an action that directly results in stress relaxation, creep, and growth of the wall; remodeling refers to a chemical modification of the wall, without the implication that it causes wall loosening. For instance, the action of xyloglucan endotransglucosylase to cut and ligate non-load-bearing xyloglucans is remodeling, whereas the action of expansins that results in cell wall creep is loosening.
Stress relaxation versus creep	When a growing cell wall is held at a constant tensile force, it extends by a slow, time-dependent, and irreversible process (creep), largely dependent on continued wall loosening. Stress relaxation is the flip side of this process: when a stretched wall is locked to a constant length, the tensile stress in the wall decays as polymers rearrange themselves to a lower energy state.
Stress	Force per area, often given in units of megapascals; tensile stresses are discussed most often, but compressive and shear stresses also occur in cell walls.
Strain	Fractional change in dimension of the wall (e.g., a strain of 0.1 in wall length is a 10% extension); strains may refer to length, width, thickness, area, or volume.
Modulus	A measure of wall stiffness, usually defined as the slope of the stress-versus-strain curve. There are different kinds of moduli, reflecting the different ways a stress may be applied and whether the resulting strain is reversible or not.
Compliance	The reciprocal of modulus, it is the tendency of the wall to deform under the action of an applied force.
Elastic versus plastic compliance	When a wall is pulled tight and then released, part of the resulting strain is reversible (termed elastic) and part is irreversible (termed plastic); the corresponding compliances are the ratios of strain/stress for the reversible and irreversible strains *.
Wall extensibility	Defined here as the ability of the cell wall to increase in surface area irreversibly during growth

*This operational definition hides the fact that the irreversible component of strain for plant cell walls is complex and time-dependent, and may include a delayed elastic component and a viscous component as well as a plastic component. Plasticity is generally defined as rapid and irreversible deformation when stress exceeds a threshold. However, technical definitions of plasticity have varied among authors. See 1 for additional details.

The term “wall loosening” has been used in diverse contexts: for instance, the indiscriminate breakdown of wall polymers by ammonia explosion pretreatment of biomass for biofuel production ^2^ and the oxidative scission of polysaccharides by hydroxyl radicals during seed germination, fruit softening, abscission, and defense responses ^3– 8^. Although one might first think of lytic actions as causing wall loosening, it turns out that the most potent of the natural wall-loosening catalysts—expansins—lack detectable wall lytic activity, presenting continuing enigmas about how they function at the molecular level and how the plant cell wall is structured to enable expansin-mediated wall loosening and surface expansion.

## Evolving concepts of cell wall structure

The growing cell wall is made of strong, stable, and inextensible cellulose microfibrils embedded in a hydrated matrix of polysaccharides classified as pectins and hemicelluloses ^9– 12^. Diverse proteins and proteoglycans are also present in small amounts. Concepts of how these components form a strong yet extensible wall have evolved considerably, inevitably influencing our notions of wall loosening. Fifty years ago, the growing cell wall was viewed as a mat of cellulose microfibrils embedded in an amorphous matrix that yielded plastically to the forces of cell turgor ^13, 14^. In this concept, wall loosening was thought to result from reduction of matrix viscosity by the action of lytic enzymes, but later results showed that changes in wall viscoelastic properties are not the basis for cell wall loosening and growth, at least in many contexts ^15, 16^. Nevertheless, these oversimplified notions of wall structure and wall loosening continue to exert a strong influence on current thinking.

**Figure 1.**
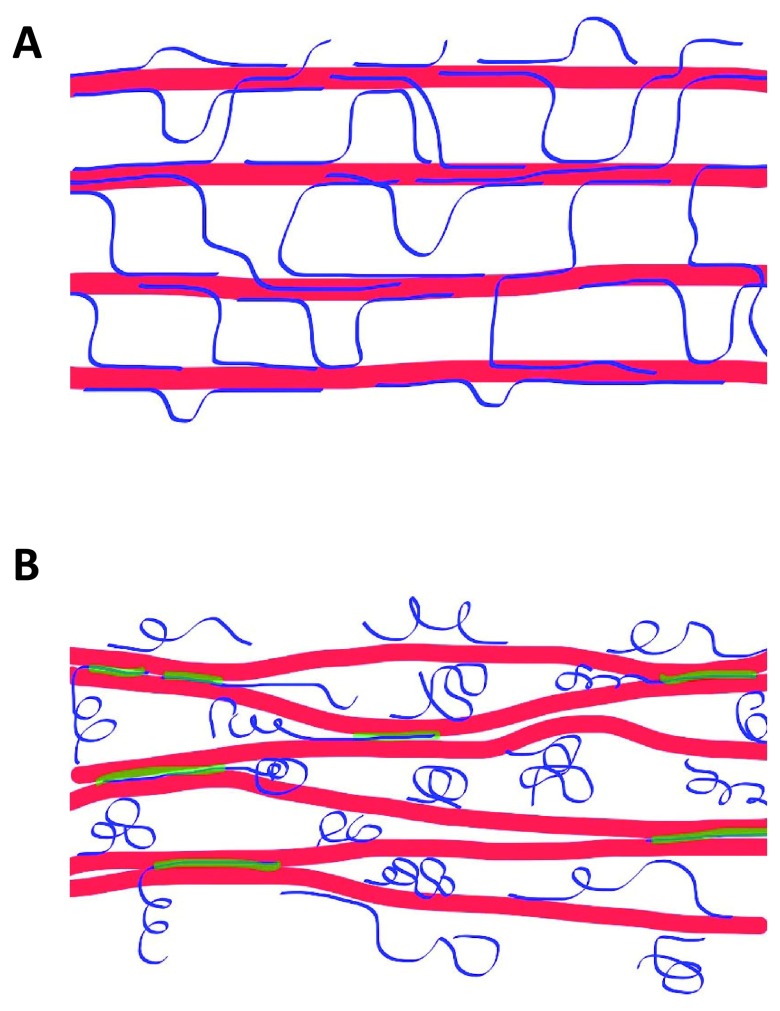
Comparison of two contemporary models of primary cell wall structure, differing in how cellulose microfibrils are mechanically connected. ( **A**) The tethered network model proposes that cellulose microfibrils (red) are well separated by matrix polysaccharides, including xyloglucans (blue) which bind to cellulose microfibrils and tether them to form a load-bearing molecular network. ( **B**) The “biomechanical hotspot” model posits limited cellulose-cellulose junctions that are bonded together by a xyloglucan-cellulose amalgam (green) with limited enzymatic accessibility. The limited frequency of these junctions means that mesoscale aspects of wall architecture and motions may predominate over nanoscale structure in limiting cell enlargement. Additionally, xyloglucan is shown in both a coiled configuration and a highly extended form, but which form predominates in cell walls is uncertain.

A major conceptual departure from this view came from the Albersheim group, who proposed that cellulose microfibrils were separated from each other by a massive macromolecule made of pectin, glycoprotein, and xyloglucan, with xyloglucan binding tightly to cellulose surfaces, thereby forming a load-bearing molecular network ^17^. Such a structure would have very different mechanical properties than the previous concept of the cell wall as a fiberglass-like structure, and suggested potential sites and mechanisms for wall loosening. When these were not substantiated, the Albersheim model was abandoned in favor of an alternative in which xyloglucan directly tethered cellulose microfibrils to form an interconnected network that was embedded in a viscous, gel-like pectin matrix ( Figure 1A) ^9, 10, 18^. This “tethered network” model puts xyloglucan in the limelight as the major target of wall loosening and has dominated discussion of primary cell walls for more than two decades. It differs from the old model of an amorphous matrix reinforced with cellulose microfibrils in that xyloglucans were viewed as direct tethers that bind tightly and extensively to cellulose surfaces, forming the sole load-bearing links between cellulose microfibrils.

Recent results, however, have weighed against the tethered network model: (a) *Arabidopsis* mutants lacking xyloglucan have a relatively minor growth phenotype ^19– 21^, showing that xyloglucan is not essential for a functional, growing cell wall. (b) Nuclear magnetic resonance (NMR) results showed that xyloglucan-cellulose interactions are not as prevalent as expected from the model ^22^, but that pectin-cellulose interactions are much more abundant than expected ^23, 24^. (c) Digestion of cell walls with xyloglucan-cutting enzymes did not reduce wall strength or cause cell wall extension, despite the prediction of the tethered network model ^25, 26^.

**Figure 2.**
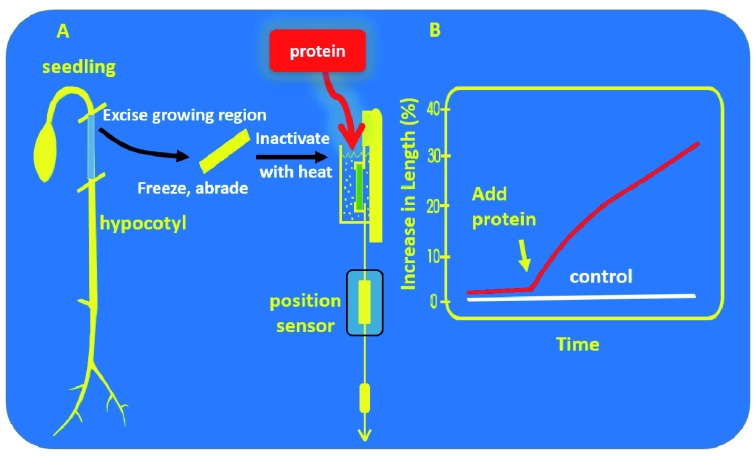
Schematic drawing of the procedure for measuring cell wall creep in a constant force extensometer. ( **A**) A cell wall sample is prepared from a growing plant tissue, such as a young hypocotyl from a seedling, and clamped at constant force in an apparatus that continuously measures changes in sample length. The buffer surrounding the sample can be exchanged for one containing a candidate wall-loosening protein. ( **B**) Time course for change in length, using a typical response to α-expansin as an example. The cell wall creep measured in this device is dependent on continuous wall loosening by expansins or other proteins, and thus mimics aspects of cell wall enlargement in living cells.

A revised concept of wall structure emerged from a study that made use of the method outlined in Figure 2 to test the ability of substrate-specific endoglucanases to induce cell wall creep ^26^. Enzymes that cut only xyloglucan or only cellulose did not induce cell wall creep, whereas endoglucanases able to cut both xyloglucan and cellulose did induce creep. A family-12 glycosyl hydrolase (GH12) named Cel12A, from the fungus *Trichoderma reesei*, was ** particularly effective at causing cell wall creep. Enigmatically, the combination of xyloglucan-specific and cellulose-specific enzymes—both GH12 enzymes and structurally similar to Cel12A—lacked wall-loosening action. This puzzling result was interpreted to mean that walls were loosened only when a relatively inaccessible amalgam containing xyloglucan and cellulose was digested by a single enzyme with both xyloglucanase and cellulase activities. To account for the ineffectiveness of two separate enzymes with distinct substrate specificities, the amalgam was hypothesized to be buried within tight junctions between two or more cellulose microfibrils. These and other results led to the revised concept depicted in Figure 1B, in which wall extensibility is controlled at limited sites (“biomechanical hotspots”) of close contact between cellulose microfibrils ^26^.

Subsequent results support the concept that cellulose-cellulose contacts may be important for wall mechanics. Making use of advances in atomic force microscopy (AFM), studies of never-dried primary cell walls showed the nanoscale arrangement of cellulose microfibrils and the presence of cellulose-cellulose junctions ^27, 28^. The ability to image cell walls under water is a key advantage of AFM compared with high-resolution scanning electron microscopy, which requires the sample to be dry, potentially causing wall polymers to coalesce. Water plays a big role in the structure and mechanics of primary cell walls ^29– 31^. Other recent work used molecular dynamics simulations to show that cellulose-cellulose junctions, glued together by a monolayer of xyloglucan, are strong enough to contribute substantially to cell wall mechanics ^32^. A clue to the potential role of the bulk of xyloglucans in the wall emerged from a recent study of an *Arabidopsis* mutant lacking xyloglucan: cellulose microfibrils were parallel to each other, whereas in the wild type they were more dispersed ^21^. This result suggests that xyloglucans may orchestrate cellulose-cellulose interactions in complex ways.

The revised model in Figure 1B does not address the potential role of direct pectin-cellulose interactions ^24^. NMR results show that pectins include both mobile and rigid chains ^23^, interpreted to mean that some pectins form a mobile gel-like milieu but that others are tightly associated with cellulose. The latter component may contribute to the cellulose-cellulose junctions or may provide a separate set of linkages between cellulose microfibrils ^29, 33^. The extent of pectin-cellulose cross-peaks in NMR cross-polarization experiments implies an interaction that is more stable than that detected by *in vitro* binding experiments ^34^, but does not demonstrate it to be load-bearing. This remains an unresolved aspect of cell wall structure. How tensile forces in the wall are transmitted between cellulose microfibrils is a key question for understanding the molecular mechanism of wall loosening because these are the connections that must be loosened for the wall to expand irreversibly. The biomechanical hotspot concept proposes that growing cell walls contain specific, built-in junctions designed for slippage and stress relaxation by the action of expansins and other wall-loosening proteins.

### Wall stress relaxation, wall loosening, and protein catalysts thereof

In biophysical terms, cell growth begins by selective loosening of the cell wall, resulting in a relaxation of wall stress; this action creates the impetus for water uptake and physical enlargement of the cell by a process in which the wall polymers slide or otherwise separate to increase wall surface area ^1, 35^. Wall loosening has been studied *in vitro* by measuring sustained cell wall extension (creep) with an extensometer, sketched in Figure 2 ^36^. Such cell wall creep mimics the sustained enlargement of cell walls during plant growth, and enabled the discovery and initial characterization of expansins ^37, 38^. This approach reduces the complexity inherent in living cells, which may manipulate wall pH, redox state, and other variables by dynamic signaling pathways ^39^.

A legion of lytic enzymes—from plants as well as from pathogens—can cleave the backbone or sidechains of wall polysaccharides, and may even digest the cell wall to the point of mechanical failure. Such enzymatic deconstruction, potentially aided by the chemical action of hydroxyl radicals, may contribute to fruit softening, organ abscission, and pathogen attack, but these lytic activities generally do not cause sustained cell wall creep ^40, 41^. Evidently, wall loosening during cell enlargement is subtler than simple breakdown of cell wall polymers. The rest of this review summarizes the action of enzymes and other wall-active proteins ascribed a wall-loosening function. The complex actions of reactive oxygen species, such as hydroxyl radicals ^42– 45^, are beyond the scope of this review. The first group of wall-loosening proteins to be discussed (expansins) have no detectable enzymatic activity, yet are the clearest examples of endogenous catalysts of plant cell wall loosening. The term “catalyst” is used here in the general sense and does not imply a change in the covalent structure of cell wall components.

## Expansins

The activity of three classes of expansins has been characterized to date: α-expansins, β-expansins, and bacterial expansins ^46, 47^. The first expansins—now identified as α-expansins—were discovered by a reconstitution approach in which protein extracts from growing plant cell walls were added to heat-inactivated cell walls clamped in a extensometer to restore their ability to extend irreversibly ( Figure 2) ^48^. The proteins induced wall creep and wall relaxation, yet they neither hydrolyzed the cell wall nor exhibited other enzyme activities ^49– 51^. Their wall-loosening activity was maximal at low pH (~4), consistent with their role in the so-called acid growth response of plants and the rapid induction of cell elongation by auxin-induced acidification of the cell wall space ^52, 53^.

Experiments with cucumber hypocotyl walls showed that α-expansins did not weaken the cell wall, as measured by mechanical (stress/strain) assays ^54^. The ability of α-expansins to induce creep without reducing wall stiffness provided additional evidence that they do not cut cell wall linkages, which would result in reduced cell wall stiffness as well as release of wall polysaccharide fragments. Other studies showed that α-expansin binding to matrix-depleted cell walls saturated at a value of approximately 1:1,000 (dry mass of protein:wall) ^50^. A recent calculation showed that this value corresponds to a spacing of approximately 200 nm between expansin binding sites within a cell wall lamella ^55^, implying that the mesoscale (between molecular and cellular scales ^56^) is the appropriate scale for understanding the mechanics of growing cell walls. Molecular-scale models of cell walls (i.e., a 50-nm cube) may be focused on too small a piece of the cell wall to capture crucial structural aspects of cell wall growth.

Application of α-expansins to living tobacco cell cultures enhanced cell growth ^57^, consistent with a host of reports in which ectopic expression of α-expansin genes likewise stimulated plant growth (reviewed in 38, 58). Wang *et al.* ^59^ used a single-cell compression assay to estimate the stiffness (elastic modulus) and the bursting force of living tomato suspension culture cells treated with α-expansin. Within a physiologically realistic extracellular pH range (4.5–6.0), α-expansin treatment did not change wall stiffness, consistent with the results cited above for cucumber hypocotyls ^54^. Curiously, a *higher* force was required to cause bursting of cells treated with α-expansin at acidic pH values (when α-expansins are most active), compared with untreated cells. The increased toughness may be a consequence of enhanced force dissipation by α-expansin-mediated wall relaxation during the compression, evidenced by higher strain at failure for cells treated with α-expansin. Apparently, the loosening action of α-expansin can result in what appears to be a tougher wall (greater mechanical energy required for failure). A lesson from this example is that different mechanical assays report on different aspects of cell wall mechanics, and what may seem at first glance to be a contradictory result may be consistent with a specific loosening mechanism.

Contrary to the above reports that α-expansin does not mechanically weaken cell walls, constitutive overexpression of an α-expansin gene in rice suspension cells resulted in a six-fold reduction in wall stiffness as measured by micro-indentation assay ^60^. It would be premature, however, to conclude that such weakening was a direct action of α-expansin, because large differences in cell size and wall composition were noted between control and the constitutive overexpressor cell lines, and because it is likely that α-expansin overexpression led to changes in wall synthesis and assembly that impacted the micro-indentation results.

A second set of plant expansins encompasses the β-expansin group, also encoded by a multigene family throughout land plants ^38^. Characterization of protein activity has been limited almost exclusively to a unique clade of β-expansins that are expressed at high levels in grass pollen ^61– 64^, and that were evolutionarily co-opted in grasses to aid penetration of the pollen tube through the grass stigma and style ^65, 66^. These proteins have drawn attention in the immunology field because they are major allergens of grass pollen; thus, their alias as “group I grass pollen allergens” ^67^. The crystallographic structure of β-expansin from maize pollen revealed a two-domain protein with domain one (D1) resembling the fold of family-45 endoglucanases (GH45 in the www.cazy.org classification system) and a second domain (D2) forming a β-sandwich with a presumptive binding function ^64^. It is notable that some of the GH45 catalytic residues are conserved in plant expansins, but a key aspartic acid residue that functions as the general base in many GH45 endoglucanases is missing, potentially accounting for the lack of hydrolytic activity.

The β-expansins in the pollen-allergen group have two properties that may not be common to the larger group of β-expansins: they selectively loosen cell walls of plants in the grass family (Poaceae), which have a wall composition distinctive from that of most land plants ^63^, and they solubilize matrix polysaccharides—arabinoxylan and homogalacturonan (HG)—found both in the cell wall and in the intercellular adhesive, or middle lamella, between cells of grasses ^68^. Solubilization of the matrix suggested a lytic action, but several tests for lytic activities gave negative results. It is relevant here to note that these polymers can be solubilized from walls by chemical extractants that do not break covalent bonds. Unlike α-expansins, pollen β-expansins greatly reduced the tensile strength of grass cell walls, at least in part by weakening the middle lamella between cells, whereas they had negligible effect on cell walls from eudicot species ^69^. These results suggest that eudicot walls lack the specific target of pollen β-expansins, or that the target has a minor mechanical role in eudicot walls. Binding studies suggested arabinoxylan (a hemicellulose) as a potential binding target ^64^; however, not all cell walls rich in arabinoxylan were loosened by pollen β-expansin ^63^, leading to the suggestion that grass cell walls have a unique cross-linking structure that is the specific target of pollen β-expansins. Further work is needed to identify this wall component and its structural role in grass cell walls. Moreover, the loosening actions of other β-expansins, outside the pollen group, have not yet been explored, in part because they have been difficult to extract from cell walls in active form ^70^ and because attempts to produce plant expansin proteins by heterologous expression have met little success.

A third group—bacterial expansins—was recognized through structural and phylogenetic approaches. The crystal structure of a *Bacillus subtilis* protein, renamed BsEXLX1 according to expansin nomenclature, was found to be homologous to the structure of pollen β-expansin ^71^. Wall extension assays showed that BsEXLX1 could induce cell wall creep, but only weakly. Like α-expansins, it did not weaken cell walls in stress/strain assays nor did it exhibit lytic activity with isolated cell wall polysaccharides or with whole cell walls as substrates, yet it weakened paper, a mat of pure cellulose fibers. In these respects, it behaved like a weak α-expansin.

Phylogenetic analysis identified expansins in a number of other bacteria that are plant pathogens ^72, 73^, evidently the result of horizontal gene transfer from plants. When expansins from *Xanthomonas campestris*, *Clavibacter michiganensis*, *Ralstonia solanacearum*, and *Aspergillus niger* (all plant pathogens) were recombinantly expressed in *Escherichia coli* and tested for their ability to induce creep of cell walls, they consistently exhibited positive but weak activity ^74^. Gene knockout experiments, recently reviewed ^46^, indicate that bacterial expansins facilitate bacterial colonization of plant surfaces. Exactly how this works is unclear because their wall-loosening activity is so weak; their specific activity is perhaps 100 times lower compared with α-expansins. We do not understand the structural basis for the high activity of α-expansins versus low activity of bacterial expansins, but the consistently low activity of the latter may have evolved to avoid plant defenses that sense cell wall integrity ^75, 76^.

**Figure 3.**
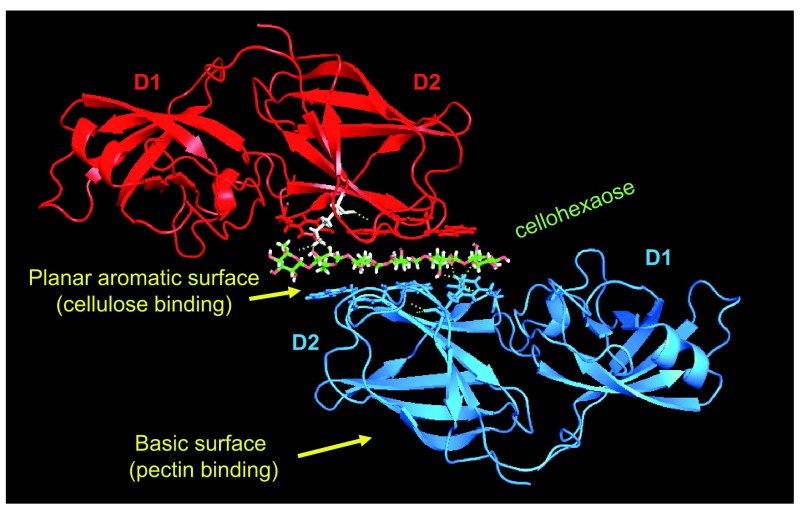
Crystallographic structure of expansin-cellulose complex (expansin from *Bacillus subtilis*). Two proteins (red and blue) in the crystallographic unit form a sandwich-like structure with cellohexaose (green), an oligosaccharide form of cellulose ^78^. The interactions with cellohexaose are mediated exclusively through the open planar surface of the second domain (D2) and depend mostly on hydrophobic interactions with three aromatic residues arranged in a spaced, linear configuration so they bind the hydrophobic face of alternating glucose residues. The sandwich-like structure probably does not form in cell walls, but it provides structural information about the interaction of expansin with cellulose surfaces. Abbreviations: D1, domain 1; D2, domain 2.

Because plant expansins have proven so difficult to express in active recombinant form, the *B. subtilis* expansin BsEXLX1 was used in place of plant expansins for extensive structure-function analysis by site-directed mutagenesis ^77^, crystallography ^78^, and NMR ^79^, combined with binding and activity assays ( Figure 3). We have learned a lot from these studies. Domain D2 proved to be the major determinant of expansin binding to plant cell walls, but two distinctive modes of binding to cellulose and to pectin were identified. Site-directed mutagenesis showed that binding to cellulose required three aromatic residues on the surface of D2 ^77^. This was confirmed and extended by crystallographic studies of protein-ligand complexes ^78^, which showed that these three residues bound alternating glucose residues in cellulose oligosaccharides, predominantly through hydrophobic interactions ( Figure 3). Cellulose binding was required for wall loosening. On the other hand, binding of BsEXLX1 to whole cell walls was dominated by electrostatic binding to acidic polysaccharides via non-conserved basic residues on the “back side” of the D2 domain. Mutagenesis of these basic residues greatly reduced total wall binding, but actually increased wall creep activity ^77^ and enhanced binding to cellulose within the wall, as detected by ^13^C solid-state NMR ^79^. Domain D1 did not bind to cellulose or whole cell walls yet was essential for activity. Site-directed mutagenesis also showed that an aspartic acid residue (Asp82) in D1 was essential for creep activity; this residue is part of the catalytic site conserved in GH45 endoglucanases and MltA-type lytic transglycosylases ^71^. This result suggests a cryptic enzymatic activity that has yet to be discovered. Pastor *et al.* ^80^ hypothesized, as an alternative idea, that electrostatic polarization on the expansin surface may mediate its wall-loosening action by weakening hydrogen bonding within cellulose. In an NMR study of BsEXLX1 targeting within complex plant cell walls, expansin was seen to bind cellulose with a different chemical shift than bulk cellulose, indicating a slightly modified configuration of the glucan chains in the cellulose target ^79^. Whether this modification was a result of expansin action is uncertain; more likely, expansin selectively binds to an altered form of cellulose. Moreover, xyloglucan was in close proximity to the binding site, which thus resembled the biomechanical hotspots described above. The lessons learned from structure-function analysis of bacterial expansin extend in part to plant expansins, but functional differences still lack structural explanations (e.g., why bacterial expansins are less active than α-expansin, and why they lack the matrix-solubilizing activity of the pollen β-expansins).

BsEXLX1 and other bacterial expansins have also drawn considerable attention as possible synergists of cellulose deconstruction by cellulases, with contradictory reports. This topic was recently reviewed ^46^, and the conclusion was that the reported synergistic actions of BsEXLX1 addition were attributable, at least in part, to non-specific protein effects that predominate at very low cellulase loadings and low cellulose conversion (~1%). To be relevant for commercial use, synergistic activity at high cellulose conversion should be demonstrated.

In summary, the three classes of expansins outlined here are similar in their two-domain structure and their ability to induce creep of plant cell walls, but their biological roles differ:

a. α-expansins mediate acid-induced extension of plant cell walls without mechanically weakening the cell walls;
b. pollen β-expansins not only cause cell wall creep but also solubilize polysaccharides in the middle lamella between the cell walls of grasses (but not other plants), thereby facilitating penetration of the pollen tube to the ovary; the physical actions of other β-expansins are almost certainly different but have yet to be documented;
c. bacterial expansins facilitate colonization of plant tissues by a mechanism yet to be established but presumably linked to their weak wall-loosening action.

## Endoglucanases and endotransglucosylases

These two classes of plant enzymes are often called wall-loosening enzymes, a point to be examined below, but first it is instructive to compare the loosening action of α-expansin with that of the fungal endoglucanase Cel12A, described above. Wall creep induced by Cel12A begins after a substantial lag, 6 to more than 60 minutes, depending on enzyme concentration ^54^, whereas with α-expansin it begins within seconds ^37^. Cel12A treatment increased the elastic and plastic compliances of cucumber hypocotyl walls, but α-expansin treatment did not. Cel12A hydrolyzed xyloglucan and cellulose, releasing fragments to the buffer, but this was not the case for α-expansin. It is possible that both of these proteins exert their wall-loosening effects at the same sites (biomechanical hotspots) but by different mechanisms: α-expansin induces slippage at these junctions, whereas Cel12A digests the junctions. But is there evidence that plant enzymes possess wall-loosening activity similar to that of Cel12A?

According to genome analyses as well as enzymatic assays, plants possess diverse wall lytic enzymes classified into numerous families ^41, 81^, two of which (GH9 and GH16) may cut β1,4-D-glucans (e.g., xyloglucan or cellulose). In the plant cell wall literature, GH16 enzymes are usually called xyloglucan endotransglucosylase/hydrolases (XTH) and are encoded by a large multigene family ^82, 83^. They cut xyloglucan and join the new reducing end to the non-reducing end of another xyloglucan (a transglucosylation) or to water (a hydrolysis). When XTH enzymes were first discovered, they were hypothesized to be wall-loosening enzymes, but subsequent experiments show them to have little or no ability to induce cell wall creep and to exert only minor effects on wall mechanics (assessed with stress/strain measurements). For instance, an XTH with strong transglucosylase activity from tomato was tested for its ability to induce wall creep or to weaken the wall as measured in stress/strain assays, with negative results ^25^. These results are consistent with results obtained with xyloglucan-specific GH12 endoglucanases ^26^, described above, showing that cutting of xyloglucan is not sufficient for cell wall loosening. Endogenous XTH activity in *Arabidopsis* hypocotyls is highest after elongation ceases ^84^, indicating a turnover or remodeling function other than wall loosening. Genetic knockout of XTH genes expressed in the *Arabidopsis* root and hypocotyl completely eliminated xyloglucan hydrolase activity, but did not result in a growth phenotype ^85^, indicating a dispensable role for growth. Consistent with this conclusion, transgenic overexpression of XTH in tomato hypocotyls did not affect hypocotyl growth ^86^ but did result in subtle changes in wall composition, accompanied by small (<5%) and inconsistent changes in mechanical extensibility. When four different XTH genes were constitutively overexpressed in *Arabidopsis*, small (~10%) increases in hypocotyl length were observed for two genes, but no effect was observed for the other two genes. In contrast, Van Sandt *et al.* ^87^ concluded that a recombinant *Selaginella* XTH caused wall loosening when applied to onion walls, but the effects were small. They observed a mechanical effect of XTH application when force was applied in the direction transverse to net cellulose orientation, but not in the direction parallel to net cellulose orientation. Their assay involved measuring wall extension immediately upon application of force; walls treated with exogenous XTH extended more rapidly than control walls during a time window of 10–30 minutes after application of the force. This result suggests that dynamical remodeling of xyloglucans in a rapidly extending wall may synergistically enhance wall extension, but it does not show that XTH itself can induce wall relaxation or creep. With this mode of action, XTH might be termed an indirect or secondary loosening agent ^88^ to differentiate its indirect action from that of a primary loosening agent that directly catalyzes cell wall creep.

Simmons *et al.* ^89^ recently reported that an enzyme in the GH16/XTH family, uniquely found in horsetail ( *Equisetum spp*.), was able to carry out an unusual transglucosylation, using cellulose as the lytic (donor) substrate and xyloglucan as the acceptor substrate. This action would be expected to form a covalent link between cellulose and xyloglucans and might result in cell wall stiffening. However, the mechanical consequences of this unusual GH16 activity have not been reported. An XTH enzyme from barley also showed activity with cellulose-like substrates, but the activity was very low ^90^.

To summarize this section, the totality of GH16 results leads me to conclude that XTH does not cause appreciable cell wall loosening, but is likely involved in xyloglucan remodeling and turnover during primary wall formation and after cell elongation has ceased. Some GH16 enzymes may stitch newly synthesized xyloglucan chains into xyloglucans already anchored in the wall, thus forming larger molecules ^55, 91^. Why such action has so little effect on wall mechanics seems puzzling—an indication that we lack a deep understanding of the structural determinants of plant cell wall mechanics.

Let us now consider plant GH9 enzymes, often called endoglucanases or cellulases. The database at www.cazy.org identifies a variety of activities for (mostly microbial) GH9 enzymes, including hydrolysis of xyloglucans, mannans, and xylans, as well as cellulose and (1,3;1,4)-β-D-glucans, so it is possible that the plant enzymes do more than cut cellulose or xyloglucan. This is consistent with their sequence diversity: phylogenetic analysis revealed more than 11 diverse GH9 clades in plants, and expression patterns indicate that they are involved in cell wall modification during fruit softening, abscission, growth, wood formation, and defense ^92– 96^. One well-studied GH9 clade includes a membrane-associated endoglucanase (called KORRIGAN) that is part of the cellulose synthesis complex and that influences the organization of cellulose in the wall ^97– 99^.

Whether plant GH9 enzymes directly cause wall relaxation and expansion is uncertain, but limited experimental results support this possibility. Two studies reported that plant GH9 enzymes can hydrolyze cellulose and xyloglucan *in vitro* ^100, 101^, so they may be able to induce cell wall creep, but this test has not been reported. In contrast to these two reports, another GH9 enzyme from tomato could cut (1,3;1,4)-β-D-glucan but was unable to cut either xyloglucan or crystalline cellulose ^102^. Overexpression of a poplar GH9 gene in *Arabidopsis* resulted in high levels of cello-oligosaccharides in the leaf, taken as evidence of cellulase action by the enzyme ^103^. Leaf growth was increased in the overexpressing lines, as was plastic compliance, measured in stress/strain assays.

In summary, the limited experimental results suggest that some plant GH9 enzymes may directly loosen the cell wall to induce stress relaxation and wall creep, but more work is needed to demonstrate direct wall-loosening activity and to test whether plants actually use these enzymes for this function *in vivo*.

## Pectin methylesterase and other pectin-modifying enzymes

Interest in the potential wall-loosening activity of pectin-modifying enzymes has increased recently ^104– 106^, in part because of puzzling results suggesting that sites of leaf initiation on shoot apical meristems are softer (lower elastic modulus) as a result of de-esterification of pectin (HG) ^107, 108^. HG is synthesized in the Golgi apparatus and delivered to the cell wall with most of the carboxyl groups blocked with methyl esters ^12^, making it resistant to the lytic action of pectate lyase and many endogalacturonases. Disruption of the normal delivery of pectin to the cell wall ^109^, or its de-esterification ^106^, leads to substantial growth defects. From studies of nuclear magnetic spin transfer within *Arabidopsis* cell walls ^22, 110^ and mechanical assays of *Arabidopsis* pectin mutants ^111^, it appears that pectins are physically entangled with xyloglucan within the wall matrix. After delivery of HG to the cell wall, methyl esters are removed by the action of pectin methylesterase (PME), encoded in plants by a large multigene family. The puzzle mentioned above stems from the contradiction with well-established results showing that de-esterified pectins *in vitro* form stiffer gels than do methyl-esterified pectins, and that pectin de-esterification *in vivo* is associated with cell wall stiffening as cells cease elongation ^112, 113^. Stiffening arises from cooperative calcium binding of contiguous carboxyl groups on two adjacent pectin chains ^114^. Thus, one would expect that regions of de-esterified HG in the meristem would be stiffer, not softer. Contrary to this expectation, reduction of PME activity by ectopic expression of PME inhibitor proteins resulted in stiffer walls, measured by micro-indentation of the plant surface ^106– 108^. At this point, the reason for the softer walls in regions rich in de-esterified pectin is unexplained. One possibility is that de-esterified pectins get cleaved into shorter chains by endogenous endogalacturonase and lyase, but there is scant evidence for this. Another possibility is that walls in the meristem lack sufficient calcium for pectic gel formation; without calcium cross-linking, the negative charges on the HG chains might cause cell wall swelling and softening. A third possibility is that manipulation of the state of pectin activates cell wall integrity sensors, activating brassinosteroid signaling ^115^ and potentially inducing many changes in cell wall composition and structure that result in altered wall mechanics. Further work will be necessary to understand these contrary associations between pectin esterification and wall stiffness. In any case, there is no evidence that PME directly causes wall stress relaxation or creep, so its action is of a different kind altogether.

## Prospectus

Recent studies are converging on the concept that the primary cell wall contains limited cellulose-cellulose junctions that are sites of initial wall loosening and stress relaxation, and that are the selective targets of expansins and potentially other wall-loosening agents. How these sites are formed is unknown; are they the result of a well-controlled cellular process or of a purely physical, stochastic interaction? Their detailed structure and spatial distribution need to be investigated, perhaps starting with novel tagging procedures. We also need to know whether plant GH9 enzymes can loosen the wall in the manner of Cel12A. The contradictory reports of PME action on cell wall properties present an unresolved puzzle, and the functional significance of extensive pectin-cellulose interactions, seen in NMR studies, needs deeper study to understand their possible significance for cell wall mechanics and growth.

## Abbreviations

AFM, atomic force microscopy; D1, expansin domain 1; D2, expansin domain 2; GH9, glycosyl hydrolase family 9; GH12, glycosyl hydrolase family 12; GH16, glycosyl hydrolase family 16; GH45, glycosyl hydrolase family 45; HG, homogalacturonan; NMR, nuclear magnetic resonance; PME, pectin methylesterase; XTH, xyloglucan endotransglycosylase/hydrolase.

## References

[1] Cosgrove DJ: Plant cell wall extensibility: connecting plant cell growth with cell wall structure, mechanics, and the action of wall-modifying enzymes. *J Exp Bot.* 2016;67(2):463–76. 10.1093/jxb/erv511 26608646

[2] Pattathil S Hahn MG Dale BE: Insights into plant cell wall structure, architecture, and integrity using glycome profiling of native and AFEX ^TM^-pre-treated biomass. *J Exp Bot.* 2015;66(14):4279–94. 10.1093/jxb/erv107 25911738 PMC4493783

[3] Fry SC Miller JG Dumville JC: A proposed role for copper ions in cell wall loosening. *Plant Soil.* 2002;247(1):57–67. 10.1023/A:1021140022082

[4] Duan J Kasper DL: Oxidative depolymerization of polysaccharides by reactive oxygen/nitrogen species. *Glycobiology.* 2011;21(4):401–9. 10.1093/glycob/cwq171 21030538 PMC3055593

[5] Jeevan Kumar SP Rajendra Prasad S Banerjee R: Seed birth to death: dual functions of reactive oxygen species in seed physiology. *Ann Bot.* 2015;116(4):663–8. 10.1093/aob/mcv098 26271119 PMC4578000

[6] Schopfer P Liszkay A: Plasma membrane-generated reactive oxygen intermediates and their role in cell growth of plants. *Biofactors.* 2006;28(2):73–81. 10.1002/biof.5520280202 17379938

[7] Müller K Linkies A Vreeburg RA: *In vivo* cell wall loosening by hydroxyl radicals during cress seed germination and elongation growth. *Plant Physiol.* 2009;150(4):1855–65. 10.1104/pp.109.139204 19493972 PMC2719145

[8] Zhang Y Chen B Xu Z: Involvement of reactive oxygen species in endosperm cap weakening and embryo elongation growth during lettuce seed germination. *J Exp Bot.* 2014;65(12):3189–200. 10.1093/jxb/eru167 24744430 PMC4071836

[9] Carpita NC Gibeaut DM: Structural models of primary cell walls in flowering plants: consistency of molecular structure with the physical properties of the walls during growth. *Plant J.* 1993;3(1):1–30. 10.1111/j.1365-313X.1993.tb00007.x 8401598

[10] Cosgrove DJ: Growth of the plant cell wall. *Nat Rev Mol Cell Biol.* 2005;6(11):850–61. 10.1038/nrm1746 16261190

[11] Scheller HV Ulvskov P: Hemicelluloses. *Annu Rev Plant Biol.* 2010;61:263–89. 10.1146/annurev-arplant-042809-112315 20192742

[12] Atmodjo MA Hao Z Mohnen D: Evolving views of pectin biosynthesis. *Annu Rev Plant Biol.* 2013;64:747–79. 10.1146/annurev-arplant-042811-105534 23451775

[13] Cleland R: Cell Wall Extension. *Annu Rev Plant Phys.* 1971;22:197–222. 10.1146/annurev.pp.22.060171.001213

[14] Probine MC Barber NF: The structure and plastic properties of the cell wall of Nitella in relation to extension growth. *Aust J Biol Sci.* 1966;19(3):439–57. Reference Source

[15] Taiz L: Plant cell expansion - Regulation of cell wall mechanical properties. *Annu Rev Plant Physiol.* 1984;35:585–657. 10.1146/annurev.pp.35.060184.003101

[16] Cosgrove DJ: Wall extensibility: its nature, measurement and relationship to plant cell growth. *New Phytol.* 1993;124(1)1–23. 10.1111/j.1469-8137.1993.tb03795.x 11537718

[17] Keegstra K Talmadge KW Bauer WD: The Structure of Plant Cell Walls: III. A Model of the Walls of Suspension-cultured Sycamore Cells Based on the Interconnections of the Macromolecular Components. *Plant Physiol.* 1973;51(1):188–97. 10.1104/pp.51.1.188 16658282 PMC367377

[18] Hayashi T: Xyloglucans in the primary cell wall. *Annu Rev Plant Phys.* 1989;40:139–68. 10.1146/annurev.pp.40.060189.001035

[19] Cavalier DM Lerouxel O Neumetzler L: Disrupting two *Arabidopsis thaliana* xylosyltransferase genes results in plants deficient in xyloglucan, a major primary cell wall component. *Plant Cell.* 2008;20(6):1519–37. 10.1105/tpc.108.059873 18544630 PMC2483363

[20] Park YB Cosgrove DJ: Changes in cell wall biomechanical properties in the xyloglucan-deficient *xxt1/xxt2* mutant of *Arabidopsis*. *Plant Physiol.* 2012;158(1):465–75. 10.1104/pp.111.189779 22108526 PMC3252101

[21] Xiao C Zhang T Zheng Y: Xyloglucan Deficiency Disrupts Microtubule Stability and Cellulose Biosynthesis in *Arabidopsis*, Altering Cell Growth and Morphogenesis. *Plant Physiol.* 2016;170(1):234–49. 10.1104/pp.15.01395 26527657 PMC4704587

[22] Dick-Perez M Wang T Salazar A: Multidimensional solid-state NMR studies of the structure and dynamics of pectic polysaccharides in uniformly ^13^C-labeled *Arabidopsis* primary cell walls. *Magn Reson Chem.* 2012;50(8):539–50. 10.1002/mrc.3836 22777793

[23] Wang T Park YB Cosgrove DJ: Cellulose-Pectin Spatial Contacts Are Inherent to Never-Dried *Arabidopsis* Primary Cell Walls: Evidence from Solid-State Nuclear Magnetic Resonance. *Plant Physiol.* 2015;168(3):871–84. 10.1104/pp.15.00665 26036615 PMC4741345

[24] Wang T Zabotina O Hong M: Pectin-cellulose interactions in the *Arabidopsis* primary cell wall from two-dimensional magic-angle-spinning solid-state nuclear magnetic resonance. *Biochemistry.* 2012;51(49):9846–56. 10.1021/bi3015532 23167456

[25] Saladié M Rose JK Cosgrove DJ: Characterization of a new xyloglucan endotransglucosylase/hydrolase (XTH) from ripening tomato fruit and implications for the diverse modes of enzymic action. *Plant J.* 2006;47(2):282–95. 10.1111/j.1365-313X.2006.02784.x 16774648

[26] Park YB Cosgrove DJ: A revised architecture of primary cell walls based on biomechanical changes induced by substrate-specific endoglucanases. *Plant Physiol.* 2012;158(4):1933–43. 10.1104/pp.111.192880 22362871 PMC3320196

[27] Zhang T Mahgsoudy-Louyeh S Tittmann B: Visualization of the nanoscale pattern of recently-deposited cellulose microfibrils and matrix materials in never-dried primary walls of the onion epidermis. *Cellulose.* 2014;21(2):853–62. 10.1007/s10570-013-9996-1

[28] Zhang T Zheng Y Cosgrove DJ: Spatial organization of cellulose microfibrils and matrix polysaccharides in primary plant cell walls as imaged by multichannel atomic force microscopy. *Plant J.* 2016;85(2):179–92. 10.1111/tpj.13102 26676644

[29] White PB Wang T Park YB: Water-polysaccharide interactions in the primary cell wall of *Arabidopsis thaliana* from polarization transfer solid-state NMR. *J Am Chem Soc.* 2014;136(29):10399–409. 10.1021/ja504108h 24984197

[30] Kim K Yi H Zamil MS: Multiscale stress-strain characterization of onion outer epidermal tissue in wet and dry states. *Am J Bot.* 2015;102(1):12–20. 10.3732/ajb.1400273 25587144

[31] Evered C Majevadia B Thompson DS: Cell wall water content has a direct effect on extensibility in growing hypocotyls of sunflower ( *Helianthus annuus* L.). *J Exp Bot.* 2007;58(12):3361–71. 10.1093/jxb/erm183 17898424

[32] Zhao Z Crespi VH Kubicki JD: Molecular dynamics simulation study of xyloglucan adsorption on cellulose surfaces: effects of surface hydrophobicity and side-chain variation. *Cellulose.* 2014;21(2):1025–39. 10.1007/s10570-013-0041-1

[33] Zykwinska A Thibault JF Ralet MC: Organization of pectic arabinan and galactan side chains in association with cellulose microfibrils in primary cell walls and related models envisaged. *J Exp Bot.* 2007;58(7):1795–802. 10.1093/jxb/erm037 17383990

[34] Zykwinska A Thibault JF Ralet MC: Competitive binding of pectin and xyloglucan with primary cell wall cellulose. *Carbohydr Polym.* 2008;74(4):957–61. 10.1016/j.carbpol.2008.05.004

[35] Cosgrove DJ: Plant Cell Growth and Elongation. *eLS.*John Wiley & Sons, Ltd.2014 10.1002/9780470015902.a0001688.pub2

[36] Durachko DM Cosgrove DJ: Measuring plant cell wall extension (creep) induced by acidic pH and by alpha-expansin. *J Vis Exp.* 2009; (25):1263. 10.3791/1263 19279553 PMC2789103

[37] McQueen-Mason S Durachko DM Cosgrove DJ: Two endogenous proteins that induce cell wall extension in plants. *Plant Cell.* 1992;4(11):1425–33. 10.1105/tpc.4.11.1425 11538167 PMC160229

[38] Cosgrove DJ: Plant expansins: diversity and interactions with plant cell walls. *Curr Opin Plant Biol.* 2015;25:162–72. 10.1016/j.pbi.2015.05.014 26057089 PMC4532548

[39] Shih HW Miller ND Dai C: The receptor-like kinase FERONIA is required for mechanical signal transduction in *Arabidopsis* seedlings. *Curr Biol.* 2014;24(16):1887–92. 10.1016/j.cub.2014.06.064 25127214

[40] Cosgrove DJ Durachko DM: Autolysis and extension of isolated walls from growing cucumber hypocotyls. *J Exp Bot.* 1994;45(Spec Iss):1711–9. 11540379 10.1093/jxb/45.special_issue.1711

[41] Franková L Fry SC: Biochemistry and physiological roles of enzymes that 'cut and paste' plant cell-wall polysaccharides. *J Exp Bot.* 2013;64(12):3519–50. 10.1093/jxb/ert201 23956409

[42] Ivanchenko MG den Os D Monshausen GB: Auxin increases the hydrogen peroxide (H _2_O _2_) concentration in tomato ( *Solanum lycopersicum*) root tips while inhibiting root growth. *Ann Bot.* 2013;112(6):1107–16. 10.1093/aob/mct181 23965615 PMC3783245

[43] Steffens B Steffen-Heins A Sauter M: Reactive oxygen species mediate growth and death in submerged plants. *Front Plant Sci.* 2013;4:179. 10.3389/fpls.2013.00179 23761805 PMC3671184

[44] Gapper C Dolan L: Control of plant development by reactive oxygen species. *Plant Physiol.* 2006;141(2):341–5. 10.1104/pp.106.079079 16760485 PMC1475470

[45] Cohen MF Gurung S Fukuto JM: Controlled free radical attack in the apoplast: a hypothesis for roles of O, N and S species in regulatory and polysaccharide cleavage events during rapid abscission by *Azolla*. *Plant Sci.* 2014;217–218:120–6. 10.1016/j.plantsci.2013.12.008 24467903 PMC3929055

[46] Georgelis N Nikolaidis N Cosgrove DJ: Bacterial expansins and related proteins from the world of microbes. *Appl Microbiol Biotechnol.* 2015;99(9):3807–23. 10.1007/s00253-015-6534-0 25833181 PMC4427351

[47] Cosgrove DJ: Loosening of plant cell walls by expansins. *Nature.* 2000;407(6802):321–6. 10.1038/35030000 11014181

[48] Cosgrove DJ: Relaxation in a high-stress environment: the molecular bases of extensible cell walls and cell enlargement. *Plant Cell.* 1997;9(7):1031–41. 10.1105/tpc.9.7.1031 9254929 PMC156977

[49] McQueen-Mason S Cosgrove DJ: Disruption of hydrogen bonding between plant cell wall polymers by proteins that induce wall extension. *Proc Natl Acad Sci U S A.* 1994;91(14):6574–8. 10.1073/pnas.91.14.6574 11607483 PMC44245

[50] McQueen-Mason SJ Cosgrove DJ: Expansin mode of action on cell walls. Analysis of wall hydrolysis, stress relaxation, and binding. *Plant Physiol.* 1995;107(1):87–100. 11536663 10.1104/pp.107.1.87 PMC161171

[51] McQueen-Mason SJ Fry SC Durachko DM: The relationship between xyloglucan endotransglycosylase and *in-vitro* cell wall extension in cucumber hypocotyls. *Planta.* 1993;190(3):327–31. 10.1007/BF00196961 7763661

[52] Rayle DL Cleland RE: The Acid Growth Theory of auxin-induced cell elongation is alive and well. *Plant Physiol.* 1992;99(4):1271–4. 10.1104/pp.99.4.1271 11537886 PMC1080619

[53] Spartz AK Ren H Park MY: SAUR Inhibition of PP2C-D Phosphatases Activates Plasma Membrane H ^+^-ATPases to Promote Cell Expansion in *Arabidopsis*. *Plant Cell.* 2014;26(5):2129–42. 10.1105/tpc.114.126037 24858935 PMC4079373

[54] Yuan S Wu Y Cosgrove DJ: A fungal endoglucanase with plant cell wall extension activity. *Plant Physiol.* 2001;127(1):324–33. 10.1104/pp.127.1.324 11553760 PMC117988

[55] Park YB Cosgrove DJ: Xyloglucan and its interactions with other components of the growing cell wall. *Plant Cell Physiol.* 2015;56(2):180–94. 10.1093/pcp/pcu204 25613914

[56] Yip S Short MP: Multiscale materials modelling at the mesoscale. *Nat Mater.* 2013;12(9):774–7. 10.1038/nmat3746 23966042

[57] Link BM Cosgrove DJ: Acid-growth response and alpha-expansins in suspension cultures of bright yellow 2 tobacco. *Plant Physiol.* 1998;118(3):907–16. 10.1104/pp.118.3.907 9808735 PMC34801

[58] Sampedro J Cosgrove DJ: The expansin superfamily. *Genome Biol.* 2005;6(12):242. 10.1186/gb-2005-6-12-242 16356276 PMC1414085

[59] Wang CX Wang L McQueen-Mason SJ: pH and expansin action on single suspension-cultured tomato ( *Lycopersicon esculentum*) cells. *J Plant Res.* 2008;121(5):527–34. 10.1007/s10265-008-0176-6 18615263

[60] Ma N Wang Y Qiu S: Overexpression of *OsEXPA8*, a root-specific gene, improves rice growth and root system architecture by facilitating cell extension. *PLoS One.* 2013;8(10):e75997. 10.1371/journal.pone.0075997 24124527 PMC3790854

[61] Cosgrove DJ Bedinger P Durachko DM: Group I allergens of grass pollen as cell wall-loosening agents. *Proc Natl Acad Sci U S A.* 1997;94(12):6559–64. 10.1073/pnas.94.12.6559 9177257 PMC21089

[62] Li LC Cosgrove DJ: Grass group I pollen allergens (beta-expansins) lack proteinase activity and do not cause wall loosening via proteolysis. *Eur J Biochem.* 2001;268(15):4217–26. 10.1046/j.1432-1327.2001.02336.x 11488915

[63] Sampedro J Guttman M Li LC: Evolutionary divergence of β-expansin structure and function in grasses parallels emergence of distinctive primary cell wall traits. *Plant J.* 2015;81(1):108–20. 10.1111/tpj.12715 25353668

[64] Yennawar NH Li LC Dudzinski DM: Crystal structure and activities of EXPB1 (Zea m 1), a beta-expansin and group-1 pollen allergen from maize. *Proc Natl Acad Sci U S A.* 2006;103(40):14664–71. 10.1073/pnas.0605979103 16984999 PMC1595409

[65] Valdivia ER Stephenson AG Durachko DM: Class B beta-expansins are needed for pollen separation and stigma penetration. *Sex Plant Reprod.* 2009;22(3):141–52. 10.1007/s00497-009-0099-y 20033435

[66] Valdivia ER Wu Y Li LC: A group-1 grass pollen allergen influences the outcome of pollen competition in maize. *PLoS One.* 2007;2(1):e154. 10.1371/journal.pone.0000154 17225858 PMC1764715

[67] Laffer S Duchene M Reimitzer I: Common IgE-epitopes of recombinant *Phl p* I, the major timothy grass pollen allergen and natural group I grass pollen isoallergens. *Mol Immunol.* 1996;33(4–5):417–26. 10.1016/0161-5890(95)00152-2 8676893

[68] Tabuchi A Li LC Cosgrove DJ: Matrix solubilization and cell wall weakening by β-expansin (group-1 allergen) from maize pollen. *Plant J.* 2011;68(3):546–59. 10.1111/j.1365-313X.2011.04705.x 21749508

[69] Li LC Bedinger PA Volk C: Purification and characterization of four beta-expansins (Zea m 1 isoforms) from maize pollen. *Plant Physiol.* 2003;132(4):2073–85. 10.1104/pp.103.020024 12913162 PMC181291

[70] Lee Y Choi D: Biochemical properties and localization of the beta-expansin OsEXPB3 in rice ( *Oryza sativa* L.). *Mol Cells.* 2005;20(1):119–26. 16258250

[71] Kerff F Amoroso A Herman R: Crystal structure and activity of *Bacillus subtilis* YoaJ (EXLX1), a bacterial expansin that promotes root colonization. *Proc Natl Acad Sci U S A.* 2008;105(44):16876–81. 10.1073/pnas.0809382105 18971341 PMC2579346

[72] Nikolaidis N Doran N Cosgrove DJ: Plant expansins in bacteria and fungi: evolution by horizontal gene transfer and independent domain fusion. *Mol Biol Evol.* 2014;31(2):376–86. 10.1093/molbev/mst206 24150040

[73] Li Y Darley CP Ongaro V: Plant expansins are a complex multigene family with an ancient evolutionary origin. *Plant Physiol.* 2002;128(3):854–64. 10.1104/pp.010658 11891242 PMC152199

[74] Georgelis N Nikolaidis N Cosgrove DJ: Biochemical analysis of expansin-like proteins from microbes. *Carbohydr Polym.* 2014;100:17–23. 10.1016/j.carbpol.2013.04.094 24188833

[75] Höfte H: The yin and yang of cell wall integrity control: brassinosteroid and FERONIA signaling. *Plant Cell Physiol.* 2015;56(2):224–31. 10.1093/pcp/pcu182 25481004

[76] Hamann T: The plant cell wall integrity maintenance mechanism-concepts for organization and mode of action. *Plant Cell Physiol.* 2015;56(2):215–23. 10.1093/pcp/pcu164 25416836

[77] Georgelis N Tabuchi A Nikolaidis N: Structure-function analysis of the bacterial expansin EXLX1. *J Biol Chem.* 2011;286(19):16814–23. 10.1074/jbc.M111.225037 21454649 PMC3089525

[78] Georgelis N Yennawar NH Cosgrove DJ: Structural basis for entropy-driven cellulose binding by a type-A cellulose-binding module (CBM) and bacterial expansin. *Proc Natl Acad Sci U S A.* 2012;109(37):14830–5. 10.1073/pnas.1213200109 22927418 PMC3443152

[79] Wang T Park YB Caporini MA: Sensitivity-enhanced solid-state NMR detection of expansin's target in plant cell walls. *Proc Natl Acad Sci U S A.* 2013;110(41):16444–9. 10.1073/pnas.1316290110 24065828 PMC3799313

[80] Pastor N Dávila S Pérez-Rueda E: Electrostatic analysis of bacterial expansins. *Proteins.* 2015;83(2):215–23. 10.1002/prot.24718 25388639

[81] Sharma R Cao P Jung KH: Construction of a rice glycoside hydrolase phylogenomic database and identification of targets for biofuel research. *Front Plant Sci.* 2013;4:330. 10.3389/fpls.2013.00330 23986771 PMC3752443

[82] Hara Y Yokoyama R Osakabe K: Function of xyloglucan endotransglucosylase/hydrolases in rice. *Ann Bot.* 2014;114(6):1309–18. 10.1093/aob/mct292 24363334 PMC4195539

[83] Eklöf JM Brumer H: The *XTH* gene family: an update on enzyme structure, function, and phylogeny in xyloglucan remodeling. *Plant Physiol.* 2010;153(2):456–66. 10.1104/pp.110.156844 20421457 PMC2879796

[84] Miedes E Suslov D Vandenbussche F: Xyloglucan endotransglucosylase/hydrolase (XTH) overexpression affects growth and cell wall mechanics in etiolated *Arabidopsis* hypocotyls. *J Exp Bot.* 2013;64(8):2481–97. 10.1093/jxb/ert107 23585673

[85] Kaewthai N Gendre D Eklöf JM: Group III-A *XTH* genes of Arabidopsis encode predominant xyloglucan endohydrolases that are dispensable for normal growth. *Plant Physiol.* 2013;161(1):440–54. 10.1104/pp.112.207308 23104861 PMC3532273

[86] Miedes E Zarra I Hoson T: Xyloglucan endotransglucosylase and cell wall extensibility. *J Plant Physiol.* 2011;168(3):196–203. 10.1016/j.jplph.2010.06.029 20828871

[87] Van Sandt VS Suslov D Verbelen JP: Xyloglucan endotransglucosylase activity loosens a plant cell wall. *Ann Bot.* 2007;100(7):1467–73. 10.1093/aob/mcm248 17916584 PMC2759230

[88] Cosgrove DJ: Enzymes and other agents that enhance cell wall extensibility. *Annu Rev Plant Physiol Plant Mol Biol.* 1999;50:391–417. 10.1146/annurev.arplant.50.1.391 11541953

[89] Simmons TJ Mohler KE Holland C: Hetero-trans-β-glucanase, an enzyme unique to *Equisetum* plants, functionalizes cellulose. *Plant J.* 2015;83(5):753–69. 10.1111/tpj.12935 26185964 PMC4950035

[90] Hrmova M Farkas V Harvey AJ: Substrate specificity and catalytic mechanism of a xyloglucan xyloglucosyl transferase HvXET6 from barley ( *Hordeum vulgare* L.). *FEBS J.* 2009;276(2):437–56. 10.1111/j.1742-4658.2008.06791.x 19076217

[91] Thompson JE Smith RC Fry SC: Xyloglucan undergoes interpolymeric transglycosylation during binding to the plant cell wall *in vivo*: Evidence from ^13^C/ ^3^H dual labelling and isopycnic centrifugation in caesium trifluoroacetate. *Biochem J.* 1997;327:699–708. Reference Source 9581545 10.1042/bj3270699 PMC1218846

[92] Urbanowitcz B: Glycoside Hydrolase Family 9/Plant endoglucanases.In: Wilson DB, editor. CAZYpedia, accessed 21 September2015 Reference Source

[93] Libertini E Li Y McQueen-Mason SJ: Phylogenetic analysis of the plant endo-beta-1,4-glucanase gene family. *J Mol Evol.* 2004;58(5):506–15. 10.1007/s00239-003-2571-x 15170254

[94] Urbanowicz BR Bennett AB Del Campillo E: Structural organization and a standardized nomenclature for plant endo-1,4-beta-glucanases (cellulases) of glycosyl hydrolase family 9. *Plant Physiol.* 2007;144(4):1693–6. 10.1104/pp.107.102574 17687051 PMC1949884

[95] Buchanan M Burton RA Dhugga KS: Endo-(1,4)-β-glucanase gene families in the grasses: temporal and spatial co-transcription of orthologous genes. *BMC Plant Biol.* 2012;12:235. 10.1186/1471-2229-12-235 23231659 PMC3557191

[96] Du Q Wang L Yang X: Populus endo- *β*-1,4-glucanases gene family: genomic organization, phylogenetic analysis, expression profiles and association mapping. *Planta.* 2015;241(6):1417–34. 10.1007/s00425-015-2271-y 25716095

[97] McNamara JT Morgan JL Zimmer J: A molecular description of cellulose biosynthesis. *Annu Rev Biochem.* 2015;84:895–921. 10.1146/annurev-biochem-060614-033930 26034894 PMC4710354

[98] Vain T Crowell EF Timpano H: The Cellulase KORRIGAN Is Part of the Cellulose Synthase Complex. *Plant Physiol.* 2014;165(4):1521–32. 10.1104/pp.114.241216 24948829 PMC4119035

[99] Lei L Zhang T Strasser R: The *jiaoyao1* Mutant Is an Allele of *korrigan1* That Abolishes Endoglucanase Activity and Affects the Organization of Both Cellulose Microfibrils and Microtubules in *Arabidopsis*. *Plant Cell.* 2014;26(6):2601–16. 10.1105/tpc.114.126193 24963054 PMC4114954

[100] Ohmiya Y Takeda T Nakamura S: Purification and properties of wall-bound endo-1, 4-beta-glucanase from suspension-cultured poplar cells. *Plant Cell Physiol.* 1995;36(4):607–14. 7640890

[101] Yoshida K Komae K: A rice family 9 glycoside hydrolase isozyme with broad substrate specificity for hemicelluloses in type II cell walls. *Plant Cell Physiol.* 2006;47(11):1541–54. 10.1093/pcp/pcl020 17056618

[102] Urbanowicz BR Catalá C Irwin D: A tomato endo-beta-1,4-glucanase, SlCel9C1, represents a distinct subclass with a new family of carbohydrate binding modules (CBM49). *J Biol Chem.* 2007;282(16):12066–74. 10.1074/jbc.M607925200 17322304

[103] Park YW Tominaga R Sugiyama J: Enhancement of growth by expression of poplar cellulase in *Arabidopsis thaliana*. *Plant J.* 2003;33(6):1099–106. 10.1046/j.1365-313X.2003.01696.x 12631333

[104] Peaucelle A Braybrook S Höfte H: Cell wall mechanics and growth control in plants: the role of pectins revisited. *Front Plant Sci.* 2012;3:121. 10.3389/fpls.2012.00121 22685449 PMC3368173

[105] Wolf S Greiner S: Growth control by cell wall pectins. *Protoplasma.* 2012;249(Suppl 2):S169–75. 10.1007/s00709-011-0371-5 22215232

[106] Peaucelle A Wightman R Höfte H: The Control of Growth Symmetry Breaking in the *Arabidopsis* Hypocotyl. *Curr Biol.* 2015;25(13)1746–52. 10.1016/j.cub.2015.05.022 26073136

[107] Peaucelle A Braybrook SA Le Guillou L: Pectin-induced changes in cell wall mechanics underlie organ initiation in *Arabidopsis*. *Curr Biol.* 2011;21(20):1720–6. 10.1016/j.cub.2011.08.057 21982593

[108] Braybrook SA Peaucelle A: Mechano-chemical aspects of organ formation in *Arabidopsis thaliana*: the relationship between auxin and pectin. *PLoS One.* 2013;8(3):e57813. 10.1371/journal.pone.0057813 23554870 PMC3595255

[109] Zhu C Ganguly A Baskin TI: The fragile Fiber1 kinesin contributes to cortical microtubule-mediated trafficking of cell wall components. *Plant Physiol.* 2015;167(3):780–92. 10.1104/pp.114.251462 25646318 PMC4348757

[110] Dick-Pérez M Zhang Y Hayes J: Structure and interactions of plant cell-wall polysaccharides by two- and three-dimensional magic-angle-spinning solid-state NMR. *Biochemistry.* 2011:50(6):989–1000. 10.1021/bi101795q 21204530

[111] Abasolo W Eder M Yamauchi K: Pectin may hinder the unfolding of xyloglucan chains during cell deformation: implications of the mechanical performance of *Arabidopsis* hypocotyls with pectin alterations. *Mol Plant.* 2009;2(5):990–9. 10.1093/mp/ssp065 19825674

[112] Goldberg R Morvan C Roland JC: Composition, Properties and Localization of Pectins in Young and Mature Cells of the Mung Bean Hypocotyl. *Plant Cell Physiol.* 1986;27(3):417–29. Reference Source

[113] Zhao Q Yuan S Wang X: Restoration of mature etiolated cucumber hypocotyl cell wall susceptibility to expansin by pretreatment with fungal pectinases and EGTA *in vitro*. *Plant Physiol.* 2008;147(4):1874–85. 10.1104/pp.108.116962 18562768 PMC2492596

[114] Morris ER Powell DA Gidley MJ: Conformations and interactions of pectins. I. Polymorphism between gel and solid states of calcium polygalacturonate. *J Mol Biol.* 1982;155(4):507–16. 10.1016/0022-2836(82)90484-3 7086901

[115] Wolf S van der Does D Ladwig F: A receptor-like protein mediates the response to pectin modification by activating brassinosteroid signaling. *Proc Natl Acad Sci U S A.* 2014;111(42):15261–6. 10.1073/pnas.1322979111 25288746 PMC4210321

